# Systematic review of ocular surface treatments in the setting of thyroid eye disease

**DOI:** 10.3389/fopht.2024.1352355

**Published:** 2024-06-12

**Authors:** Anthony Stephen Wong, James G. Chelnis

**Affiliations:** ^1^ Department of Ophthalmology, John H. Stroger, Jr. Hospital of Cook County, Chicago, IL, United States; ^2^ Manhattan Face and Eye, New York, NY, United States; ^3^ New York Eye and Ear Infirmary of Mount Sinai, New York, NY, United States

**Keywords:** thyroid eye disease (TED), Graves’ disease (GD), dry eye, exposure keratopathy, ocular surface, Graves’ ophthalmopathy (GO), thyroid associated ophthalmopathy/therapy, dry eye treatment

## Abstract

**Introduction:**

Approximately 85% of patients with thyroid eye disease experience ocular surface symptoms. Although corneal exposure plays a role in inducing inflammatory changes to the ocular surface, multiple studies reveal more complexity to the abnormal tear film composition and parameters in thyroid eye disease patients including those who do not have proptosis or increased corneal exposure. Currently, a majority of cases of thyroid associated dry eye symptoms are given treatments intended for ocular surface disease arising from different etiologies.

**Methods:**

Medline via Ovid, Cochrane CENTRAL, PubMed, and Google Scholar were systematically searched for articles evaluating the efficacy of treatments for dry eye symptoms in patients with thyroid eye disease. Articles were from all geographic regions and dates ranged from inception until October 2023.

**Results:**

Seven papers ultimately met inclusion criteria and were included in this review. These papers revealed that multiple topical and non-topical treatment modalities address dry eye symptoms in thyroid eye disease and improve subjective and objective ocular surface parameters. However, due to the few studies that exist and due to disparities in sample size and study design, no overwhelming best practices were identified that could influence clinical practice.

**Conclusion:**

This systematic review identifies the current treatments that exist and highlights the clear unmet need for a large population suffering with dry eye symptoms. Ideally, further well-designed investigations into this area would target topical, non-invasive modalities to develop first line options for thyroid eye disease patients.

## Introduction

Morbidity from thyroid eye disease stems from localized ocular and orbital inflammation. The pathogenesis involves insulin-like growth factor-1 receptor (IGF-1R) and increased autoantibodies against thyrotropin receptor (TSHr). Upregulated fibroblastic activity due to increased expression of TSHr and IGF-1R in the orbit results in adipogenesis and deposition of glycosaminoglycans within local tissues (1, 2). Resulting dysfunction leads to common manifestations including eyelid retraction (92%), exophthalmos (62%), restrictive extraocular myopathy (43%), ocular pain (30%), increased lacrimation (23%), optic neuropathy (6%) and perhaps most often overlooked, symptoms related to dry eye (3).

Approximately 85% of patients with thyroid eye disease experience ocular surface symptoms (3, 4). Corneal exposure from increased palpebral fissure width induces a hyperosmolar tear film, which triggers an inflammatory cascade of interleukin (IL)-1ß, tumor necrosis factor (TNF-α), and matrix metalloproteinase (MMP)-9, which in turn, damages the ocular surface (5).

According to Gurdal et al., studies indicate worse tear film parameters such as the Ocular Surface Disease Index (OSDI), Schirmer’s test, and Tear Break Up Time (TBUT) in patients with thyroid eye disease when compared to controls “due to ocular surface inflammation rather than accelerated tear film evaporation,” proptosis, and increased corneal exposure (4). There are increased tear film concentrations of cytokines IL1-b, IL-6, IL-8, and TNF-α in patients who have thyroid eye disease without an increased palpebral fissure width (6–8). There are also abnormalities to the tear film composition of patients with Graves’ Ophthalmopathy. Some studies reveal an increased IgA to Lysozyme ratio in 33% of patients and others reveal 28% of patients with an abnormal protein composition (9, 10).

Another component to the pathogenesis of dry eye in thyroid eye disease is lacrimal gland dysfunction. Increased expression of TSH-R within the lacrimal gland leads to dacryoadenitis, which is widely prevalent and commonly underdiagnosed. Ultimately, fibrosis and lacrimal gland dysfunction develop. This, accompanied by incomplete blink and meibomian gland dropout further exacerbates dry eye symptoms (4, 11).

Currently, a majority of cases of thyroid associated dry eye symptoms are treated with treatments intended for ocular surface disease arising from different etiologies. This systematic review aims to examine the efficacy of treatments for dry eye symptoms secondary to thyroid eye disease.

## Methods

This systematic review followed the Preferred Reporting Items for Systematic Reviews and Meta-Analyses (PRISMA) guideline. A comprehensive literature search was performed using 4 electronic databases: Medline via Ovid, Cochrane CENTRAL, PubMed, and Google Scholar. The search process included both keyword free text terms and controlled vocabulary (MESH) related to the terms “Graves’ Ophthalmopathy” OR “thyroid eye disease” OR “thyroid associated ophthalmopathy” OR “Graves’ orbitopathy” AND “ocular surface disease” OR “dry eye” OR “dry eye disease” OR “exposure keratopathy” AND “treatment”. The Google Scholar search included the search terms Ocular surface disease|” “dry eye”|“exposure keratopathy”, thyroid eye disease|“Graves’ ophthalmopathy”|“Graves’ orbitopathy”|“thyroid associated ophthalmopathy”|.

### Selection criteria

The inclusion criteria included articles from inception until 2023 and no language restriction was applied. Articles describing treatments for dry eye in the setting of thyroid eye disease were considered. The primary outcomes were improvements in subjective and objective scores of the ocular surface after intervention. To ensure thorough review of all relevant papers, acceptable ocular surface changes could be reported on both a subjective scale or an objective standardized scale for ocular surface parameters. Articles from multiple geographic regions were included for review as well as those originally written in a different language and later translated into English. Clinical trials, case-control studies, cohort studies, case reports, case series, and experimental studies were included in this study. Duplicate studies, editorials, commentaries, animal studies, and reviews were excluded.

## Results

**Figure 1:**
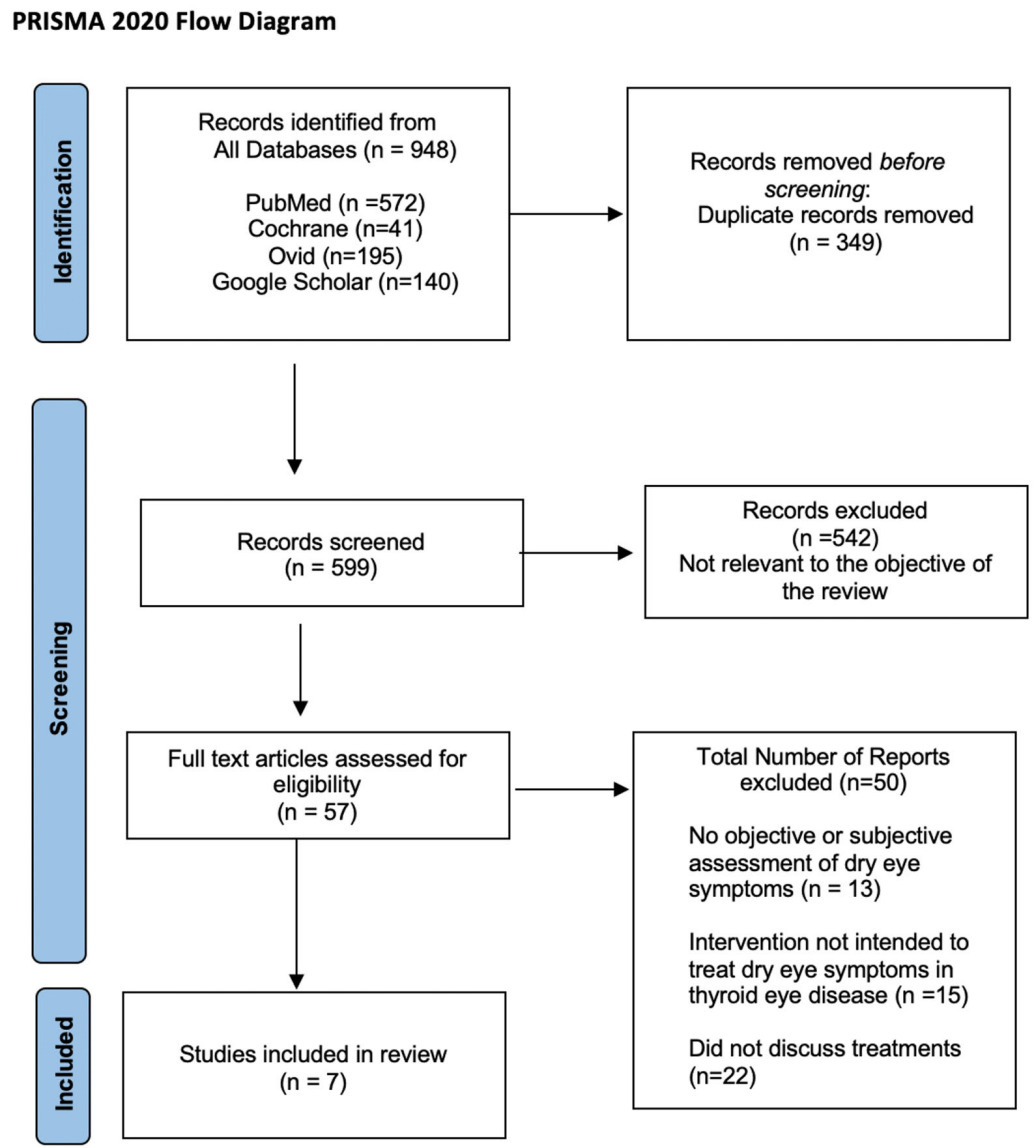
The Preferred Reporting Items for Systematic Reviews and Meta-Analyses 2020 flow diagram outlining the literature search and study selection process.

The search process yielded 948 articles. After removal of 349 duplicates, 599 remained. Title and abstract review were then performed and 542 papers were eliminated due to irrelevance to this study’s objective. The remaining 57 papers underwent a full review of the text, and 7 papers were ultimately selected and included in this review based on the criteria previously established for eligibility (**Figure 1**).

In a study of 42 patients, Altiparmak et al., revealed that 0.05% Cyclosporine A in conjunction with artificial tears improved the scores for Schirmer’s test (p=.001) and the Tear Break Up Time (TBUT) (p<.001) (12). This study included patients being treated for immune-related thyroid eye disease or with radiographic or orbital signs of thyroid eye disease with a Clinical Activity Score (CAS) of less than 4. Comparing the Cyclosporine A treatment group to the group of artificial tears alone, there was no statistically significant difference between groups. The paper suggests the addition of cyclosporine to a regimen of artificial tears is not more advantageous (12). This is in contrast to the study by Gurdal et al., which measured OSDI, Schirmer’s Test and TBUT in 12 patients (24 eyes). Subjects displayed thyroid orbitopathy-related dry eye findings, and the severity of their surface disease required an abnormal Schirmer’s test of less than 10mm in 5 minutes or TBUT less than 10 seconds in both eyes for inclusion (13). The study revealed statistically significant post-treatment improvements in dry eye parameters when compared to baseline measurements when treated with cyclosporine alone as well as improvements in the Ocular Surface Disease Index (p=.001) and TBUT (p=.001) (13). Additionally, there was less apoptosis of conjunctival epithelial cells (p=.008) and fewer mean percentage of cells that stained for MMP9 (p=.005) (13).

Sun et al., compared the efficacy between vitamin A palmitate eye gel and 0.1% sodium hyaluronate eye drops in 80 patients (80 eyes) (14). Patients included in the study were diagnosed as having dry eye syndrome in the setting of moderate to severe thyroid associated ophthalmopathy at the inactive stage. Criteria for moderate to severe determination in this study were undisclosed. Vitamin A palmitate gel statistically significantly improved the TBUT (p<.001) and overall fluorescein staining (p<.001). Although there was an improvement in Schirmer’s test results, it was not statistically significant. Overall, the effective rate of vitamin A palmitate gel treatment was 91.2%. Effective rate was defined as 2mm improvement in Schirmer test or 2 second improvement in TBUT or a 1 point decrease in fluorescein staining (0 to 12 point scale used in this study). In comparison, the 0.1% sodium hyaluronate group showed a statistically significant improvement in the OSDI and amount of fluorescein staining (p=.002). However, the improvements in Schirmer’s testing and TBUT were not statistically significant (p>.05). The overall effective rate of this treatment was 67.7%. Although both groups improved ocular surface parameters, the vitamin A palmitate group was more efficacious in treating ocular surface symptoms related to thyroid eye disease (14).

Topical rebamipide was studied in 20 patients (33 eyes) by Takahashi et al., for its use in reducing superior limbal keratoconjunctivitis (SLK) in thyroid eye disease (15). This study evaluated all patients with thyroid eye disease and SLK and excluded those wearing contact lenses or receiving treatment for TED or dry eye. Results revealed statistically significant reductions in rose bengal staining (both area and density) (p<.001), TBUT (p=.009), and presence of SLK (p<.001), but there was no improvement in Schirmer’s testing (p=.212).

Two studies examined non-topical treatment modalities. Lauer et al., studied the effect of oral montelukast and cetirizine in an open labeled trial with 12 patients (16). A statistically significant improvement was found for subjective measures on a non-standardized patient survey designed for the study of overall tearing (p<.05), overall dryness (p<.05), and overall injection (p<.05). No significant effect on diplopia or proptosis were identified.

Within a group of 15 patients, Xu et al., investigated the effect of high dose methylprednisolone (cumulative 4.5g over 12 weeks) and its effect on ocular surface measures as well as reduction in inflammatory cytokines (17). There were statistically significant reductions (p<0.05) in the OSDI and improved TBUT as well as decreases in the CAS score, lid width, and proptosis. Furthermore, there were significant reductions in IL-1β, IL-6, IL-8, TNF-α, and VEGF. However, no statistically significant change was found with respect to the Schirmer’s test, corneal fluorescein staining, or diplopia.

Two other studies investigated mechanical interventions for dry eye in thyroid eye disease. The case report by Harthan underscored the effect of an increased tear reservoir provided by the mini-scleral lens (18). This resulted in objective improvements in fluorescein staining from 2+ to trace after 3 weeks of wear as well as subjective increases in comfort. Hsu et al., performed lateral tarsoconjunctival flaps in 42 patients and showed statistically significant improvements in OSDI, TBUT, Schirmer’s test, corneal and conjunctival staining scores, and meibomian gland drop out (all p<.01) 3 months postoperatively (19). Additionally, the tear osmolarity and inflammatory markers in the tear fluid (IL-6, IL-8, IL-18 and MCP-1) were significantly improved after the lateral tarsoconjunctival flaps. All studies included may be found in the **Supplemental Materials** (**Table 1** , **Table 2**, **Table 3**).

## Discussion

### Topical treatments

Cyclosporine A, is a commonly used treatment modality for other causes of ocular surface disease (20, 21). Because there are inflammatory components to both surface disease and thyroid eye disease, cyclosporin’s function as a T-cell mediator has been explored as an adjunct for dry eye symptoms in the setting of thyroid eye disease (22). However, strong evidence does not exist to support its position as a first line treatment in the setting of thyroid eye disease, as statistically significant improvement compared to artificial tears was not observed. It is not clear why this differentiation between patient populations exists, as thyroid eye disease patients are understood to have an increased proclivity towards symptoms derived from an inflammatory etiology. Of note, these studies used 0.05% cyclosporine which is available in the United States, Canada, and 33 other countries. However, many different concentrations and formulations exist in Asia, the EU, the UK, Latin America, and Southeast Asia. Further investigation using other formulations or improved study design may aid in elucidating its use in this setting.

As can be expected, vitamin A palmitate gel produced more efficacious changes relative to 0.1% sodium hyaluronate eye drops. This is true for other dry eye patient populations as well. It also serves as a reminder that multiple sources of artificial lubricants should likely be discussed as first line agents for thyroid eye disease patients, in order to optimize treatment rapidly. Unfortunately, this increased relative efficacy of gel remains one of very few evidence-based treatments our review was able to unearth. Nonetheless, this article provides support for prioritizing Vitamin A Palmitate gel in the treatment algorithm as it may provide longer moisture coverage and thus, improve ocular surface symptoms in the setting of increased inflammation or a widened palpebral fissure due to thyroid eye disease (14).

Available in Asia, but not yet approved by the FDA in the United States, topical rebamipide improves superior limbal keratoconjunctivitis as well as ocular surface scores. There is a theoretical advantage conferred by using rebamipide when compared to other topical treatments for dry eye symptoms due to its anti-inflammatory effect and ability to increase mucin production in the cornea and conjunctiva (15). The increase in basal mucin production serves in a manner separate from replacement, which is typically the goal of other interventions. Initial positive results of this review indicate that topical rebamipide may serve as an efficacious early treatment to alleviate dry eye symptoms as a result of thyroid eye disease. However, because of the few studies available, more trials specifically focused on the thyroid eye disease population are needed to confirm the results found in this review.

### Non-topical treatments

The antihistaminic effects of oral montelukast and cetirizine on the inflammatory component of patients with thyroid eye disease remains an area of further investigation. Although the study in this review revealed positive relief of symptoms, further investigation could aid in assessing the efficacy of these treatment modalities on their own (16). Partially, this is due to limitations in study design. Future studies should also elucidate if antihistamines may serve as an adjunct to lubricating eye drops given that the side effect profile is generally well-tolerated.

Although IV methylprednisolone may decrease the inflammatory component of the tear film and improve ocular surface disease parameters, it is not a suitable treatment for all patients and is not a recognized first-line treatment in patients whose presentations are limited to ocular surface findings. Adverse side effects not limited to acute psychosis, hyperglycemia, memory impairment, hypertension, and arrhythmias should be considered prior to administration (23). Additionally, topical treatments would likely be administered as first line treatment for mild ocular surface symptoms related to thyroid eye disease (17). This does, however, serve as evidence of surface improvement that may guide treatment for patients who have more severe thyroid eye disease and find little therapeutic benefit with topical treatments. Investigations using topical corticosteroids were not made in this manuscript or otherwise in the reviewed literature.

### Mechanical interventions

Scleral contact lenses provide relief and improve the ocular surface in a variety of circumstances ranging from keratoconus to refractory ocular surface disease (24, 25). The positive results found in this review provide insight into an alternative mechanical method of moisture retention and surface protection that is underutilized in the treatment of ocular surface symptoms in thyroid eye disease. Although larger sample sizes and further investigations would be needed to validate its efficacy, initial evidence suggests it may be a useful option for corneal surface rehabilitation.

This review found lateral tarsoconjunctival flap surgery to improve dry eye parameters and decrease inflammatory markers in the tear film, but this surgical option should be reserved for patients suffering from refractory dry eye in thyroid eye disease (19). Less invasive measures focusing on tear film optimization and decreased inflammation should be trialed first, especially in patients with mild to moderate disease. However, this study provides evidence to support the next step in augmenting therapy in patients who are refractory to first line agents.

### Implications for research

Since 85% of patients with thyroid eye disease suffer from dry eye symptoms, there is a clear and large at-need population suffering with these symptoms (3, 4). The results of the systematic review highlight the paucity in research and emphasize the need for further investigation. Studies should ideally target topical, non-invasive modalities to further develop first line options for this class of patients.

Despite the fact that ocular surface symptoms present in the majority of thyroid eye disease patients and that these patients can present with evaporative dry eye changes, more research is needed to confirm that dry eye syndrome treatments have similar efficacy in the setting of thyroid eye disease. There are significant compositional and inflammatory changes to the tear film of patients with thyroid eye disease (19). These gaps in our understanding coupled with apparent immunologic complexities represent an unaddressed opportunity for research and need for an improved treatment algorithm based on evidence.

Very few manuscripts comparing common first line treatments for thyroid ophthalmopathy-related dry eye symptoms were identified in the published literature. Additionally, each study presented a limited sample size. None of the identified studies compared thyroid eye disease patients to those with a primary diagnosis of dry eye syndrome. Possible comparisons could include topical agents that include hydroxypropyl guar, cellulose, hyaluronic acid, polyethylene glycol, polyvinyl alcohol, carbomers, autologous serum tears, and lipids such as triglycerides, phospholipids, and castor oil. The development of pertinent treatment algorithms would also benefit from studies which address inflammatory pathways with corticosteroids, cyclosporine, tacrolimus, and tetracyclines. Physical interventions could be studied separately like lid taping, moisture chamber goggles, therapeutic contact lenses, and punctal plugs.

Other non-invasive modalities like intense pulsed therapy (IPL) have demonstrated their therapeutic utility, especially in those with refractory dry eye symptoms (26, 27). Although the mechanism is not fully understood, IPL has been postulated to convert light energy to heat and thrombose unwanted vasculature, decreasing blood flow and delivery of inflammatory mediators (26, 27). These types of therapies show promise given their longer therapeutic duration and positive cumulative effect.

There may be additional value in developing treatment algorithms that discriminate between acute and chronic thyroid eye disease presentations, as distinct phases in thyroid eye disease have been described. This is not seen in dry eye syndrome. Further studies should also be cognizant of occult thyroid eye disease and the mislabeling of patients with dry eye syndrome.

### Limitations

The study designs of the papers were not standardized and levels of evidence ranged from case reports to randomized controlled trials. Some treatment groups were combined rather than isolated, which may have influenced outcomes. Many studies featured a small sample size, and patient characteristics for inclusion varied greatly. Although many of the papers utilized similar measurement scales of dry eye severity like the TBUT, OSDI, and Schirmer’s Test, many did not quantitatively include the CAS score or severity of the thyroid eye disease of the patients included in the studies. This discrepancy in baseline severity would likely alter the outcomes of first line treatments, especially in more severe patients. These treatments were generally well tolerated, with the prevailing adverse effects being eye irritation or burning (4). The studies did not mention any significant alteration of outcomes as a result of adverse effects, and given the very few instances, it is possible that adverse effects were underreported.

## Conclusion

The aim of this systematic review was to provide information about the efficacy of current treatments for ocular surface symptoms in the setting of thyroid eye disease. To the best of our knowledge, this is the first systematic review that addresses this question and it clearly identified the need for additional investigation into this topic. Many treatment modalities remain uninvestigated, such as punctal plugs, intense pulsed light, radiofrequency, topical corticosteroids, topical NSAIDs, meibomian gland expression or probing, among many others.

As a result, we are unable to identify clear best practices for the treatment of ocular surface symptoms in thyroid eye disease patients. Therefore, no implication can be made to influence clinical practice. Further well-designed prospective studies that isolate treatment regimens and clearly characterize thyroid eye disease presentations, perhaps using clinical activity scores or objective physical exam findings, such as amount of eyelid retraction, are required for optimizing treatment algorithms for thyroid eye disease patients.

## Data availability statement

The original contributions presented in the study are included in the article/**Supplementary Material**. Further inquiries can be directed to the corresponding author.

## Author contributions

AW: Conceptualization, Investigation, Writing – original draft, Writing – review & editing. JC: Conceptualization, Investigation, Supervision, Writing – original draft, Writing – review & editing.

## References

[1] Braun TL Bhadkamkar MA Jubbal KT Weber AC Marx DP . Orbital decompression for thyroid eye disease. Semin Plast Surg. (2017) 31:40–5. doi: 10.1055/s-0037-1598192 PMC5330789 28255288

[2] Khoo TK Bahn RS . Pathogenesis of Graves’ ophthalmopathy: the role of autoantibodies. Thyroid. (2007) 17:1013–8. doi: 10.1089/thy.2007.0185 PMC3902014 17935483

[3] Coulter I Frewin S Krassas GE Perros P . Psychological implications of Graves’ orbitopathy. Eur J Endocrinol. (2007) 157:127–31. doi: 10.1530/EJE-07-0205 17656589

[4] Gürdal C Saraç O Genç I Kırımlıoğlu H Takmaz T Can I . Ocular surface and dry eye in Graves’ disease. Curr Eye Res. (2011) 36:8–13. doi: 10.3109/02713683.2010.526285 21174592

[5] Gilbard JP Farris RL . Ocular surface drying and tear film osmolarity in thyroid eye disease. Acta Ophthalmol. (1983) 61:108–16. doi: 10.1111/j.1755-3768.1983.tb01401.x 6687972

[6] Huang D Xu N Song Y Wang P Yang H . Inflammatory cytokine profiles in the tears of thyroid-associated ophthalmopathy. Graefes Arch Clin Exp Ophthalmol. (2012) 250:619–25. doi: 10.1007/s00417-011-1863-x 22124787

[7] Ujhelyi B Gogolak P Erdei A Nagy V Balazs E Rajnavolgyi E . Graves’ Orbitopathy results in profound changes in tear composition: A study of plasminogen activator inhibitor-1 and seven cytokines. Thyroid. (2012) 22:407–14. doi: 10.1089/thy.2011.0248 22385289

[8] Iskeleli G Karakoc Y Abdula A . Tear film osmolarity in patients with thyroid ophthalmopathy. Jpn J Ophthalmol. (2008) 52:323–6. doi: 10.1007/s10384-008-0545-7 18773272

[9] Khalil HA De Keizer RJ Bodelier VM Kijlstra A . Secretory IgA and lysozyme in tears of patients with Graves’ ophthalmopathy. Doc Ophthalmol. (1989) 72:329–34. doi: 10.1007/BF00153500 2625093

[10] Khalil HA de Keizer RJ Kijlstra A . Analysis of tear proteins in Graves’ ophthalmopathy by high performance liquid chromatography. Am J Ophthalmol. (1988) 106:186–90. doi: 10.1016/0002-9394(88)90832-X 3400761

[11] Park J Baek S . Dry eye syndrome in thyroid eye disease patients: The role of increased incomplete blinking and Meibomian gland loss. Acta Ophthalmol. (2019) 97:e800–6. doi: 10.1111/aos.14000 30593716

[12] Altiparmak UE Acar DE Ozer PA Emec SD Kasim R Ustun H . Topical cyclosporine A for the dry eye findings of thyroid orbitopathy patients. Eye (Lond). (2010) 24:1044–50. doi: 10.1038/eye.2009.246 19834507

[13] Gürdal C Genç I Saraç O Gönül I Takmaz T Can I . Topical cyclosporine in thyroid orbitopathy-related dry eye: clinical findings, conjunctival epithelial apoptosis, and MMP-9 expression. Curr Eye Res. (2010) 35:771–7. doi: 10.3109/02713683.2010.490320 20795858

[14] Sun R Yang M Lin C Wu Y Sun J Zhou H . A clinical study of topical treatment for thyroid-associated ophthalmopathy with dry eye syndrome. BMC Ophthalmol. (2023) 23:72. doi: 10.1186/s12886-023-02805-8 36803227 PMC9940084

[15] Takahashi Y Ichinose A Kakizaki H . Topical rebamipide treatment for superior limbic keratoconjunctivitis in patients with thyroid eye disease. Am J Ophthalmol. (2014) 157:807–12. doi: 10.1016/j.ajo.2013.12.027 24412123

[16] Lauer SA Silkiss RZ McCormick SA . Oral montelukast and cetirizine for thyroid eye disease. Ophthalmic Plast Reconstr Surg. (2008) 24:257–61. doi: 10.1097/IOP.0b013e318177ebac 18645426

[17] Xu N Cui Y Fu D Sun F . Tear inflammatory cytokines and ocular surface changes in patients with active thyroid eye disease treated with high-dose intravenous glucocorticoids. J Endocrinol Invest. (2020) 43:901–10. doi: 10.1007/s40618-019-01174-8 31927748

[18] Harthan JS . Therapeutic use of mini-scleral lenses in a patient with Graves’ ophthalmopathy. J Optom. (2014) 7:62–6. doi: 10.1016/j.optom.2012.11.002 PMC3938744 24646903

[19] Hsu CK Hsieh MW Chang HC Chen YH Chien KH . Improvement of ocular surface disease by lateral tarsoconjunctival flap in thyroid-associated orbitopathy patients with lid retraction. J Pers Med. (2022) 12:802. doi: 10.3390/jpm12050802 35629224 PMC9146611

[20] Sall K Stevenson OD Mundorf TK Reis BL . Two multicenter, randomized studies of the efficacy and safety of cyclosporine ophthalmic emulsion in moderate to severe dry eye disease. CsA phase 3 study group. Ophthalmology. (2000) 107:631–9. doi: 10.1016/s0161-6420(99)00176-1 10768324

[21] Zhou XQ Wei RL . Topical cyclosporine A in the treatment of dry eye: a systematic review and meta-analysis. Cornea. (2014) 33:760–7. doi: 10.1097/ICO.0000000000000123 24815112

[22] Periman LM Mah FS Karpecki PM . A review of the mechanism of action of cyclosporine A: the role of cyclosporine A in dry eye disease and recent formulation developments. Clin Ophthalmol. (2020) 14:4187–200. doi: 10.2147/OPTH.S279051 PMC7719434 33299295

[23] Sabir S Werth VP . Pulse glucocorticoids. Dermatol Clin. (2000) 18:437–46, viii-ix. doi: 10.1016/s0733-8635(05)70192-3 10943539

[24] La Porta Weber S Becco de Souza R Gomes JÁP Hofling-Lima AL . The use of the esclera scleral contact lens in the treatment of moderate to severe dry eye disease. Am J Ophthalmol. (2016) 163:167–173.e1. doi: 10.1016/j.ajo.2015.11.034 26701271

[25] Moon J Lee SM Hyon JY Kim MK Oh JY Choi HJ . Large diameter scleral lens benefits for Asians with intractable ocular surface diseases: a prospective, single-arm clinical trial. Sci Rep. (2021) 11:2288. doi: 10.1038/s41598-021-82010-z 33504920 PMC7840975

[26] Vegunta S Patel D Shen JF . Combination therapy of intense pulsed light therapy and meibomian gland expression (IPL/MGX) can improve dry eye symptoms and meibomian gland function in patients with refractory dry eye: A retrospective analysis. Cornea. (2016) 35:318–22. doi: 10.1097/ICO.0000000000000735 26785301

[27] Craig JP Chen YH Turnbull PR . Prospective trial of intense pulsed light for the treatment of meibomian gland dysfunction. Invest Ophthalmol Vis Sci. (2015) 56:1965–70. doi: 10.1167/iovs.14-15764 25678687

